# Reinforcement Learning-Enhanced Botnet Defense System in Grid Topology Networks Using the SIRO Framework

**DOI:** 10.3390/s26082517

**Published:** 2026-04-19

**Authors:** Mohd Hafizuddin Bin Kamilin, Shingo Yamaguchi, Sena Yoshioka

**Affiliations:** 1 Department of Intelligent System Engineering, National Institute of Technology, Ube College, Yamaguchi 755-8555, Japan; 2 Graduate School of Sciences and Technology for Innovation, Yamaguchi University, Yamaguchi 753-8511, Japan; f118vgw@yamaguchi-u.ac.jp

**Keywords:** botnet, cybersecurity, reinforcement learning

## Abstract

Digitalizing essential services opens up a new risk of exposing critical infrastructure to botnet infections. In a grid topology network, the neighbor-to-neighbor paths can be used by the malicious botnet to spread the infection. Previous white-hat worm launchers used heuristics and supervised learning to exterminate botnets, which demand specific conditions or a suitable dataset to be effective. Although reinforcement learning addressed these issues, it requires a longer time to train. This article proposes a framework to shorten training and improve the effectiveness of reinforcement learning. The framework applies four key principles: (1) surveying the network status with multi-tensor input, (2) removing irrelevant actions via a novel Chebyshev-based masking strategy, (3) reinforcing key actions with rewards, and (4) optimizing rewards for winning. Four reinforcement learning algorithms are implemented to evaluate the framework, which are vanilla policy gradient, deep Q-network, proximal policy optimization, and MuZero in a stylized grid topology network simulation. An ablation study indicates that the masking used in identify accounts for the majority of the improvement, whereas multi-channel in Survey alone can reduce performance without complementary masking, rewards, and optimization. With the mean winning rate improved by 49.129% and mean win efficiency improved by 118.8031% against our previous work, the framework effectiveness is confirmed in stylized simulations.

## 1. Introduction

As digitalization accelerates the implementation of Internet of Things (IoT) services in essential sectors, the growing use of connected devices creates a new challenge [1]. For example, grid topology networks are frequently utilized in wireless sensor network (WSN) applications for environmental monitoring due to their simplicity and reliability for low-powered devices [2]. Although the neighbor-to-neighbor connections that allow dynamic routing are attributed to their reliability and simplicity, their use also poses a cybersecurity risk for the spread of botnets [3,4].

Botnet infections in grid topology networks not only drain the resources, such as battery power, memory, and bandwidth, but also cause expensive downtime for patching the vulnerabilities, increased latency due to optimized routes that are not traversable, and rapid propagation potentially overwhelming containment strategies [5]. Furthermore, botnets could be remotely controlled by a botmaster to perform distributed denial-of-service (DDoS) attacks to take important services offline [6,7].

Several methods have been proposed to tackle this problem. Muthukumar et al. [8] implemented a waning-immunity infection model, which they use to develop an optimal control strategy to allocate immunization resources to reduce infections and maintain immunity. Rodríguez et al. [9] applied heuristic link pruning with the aim to isolate the infected devices and make infections die out faster. Asadi et al. [10] presented the green worm concept as a defense mechanism against infection by detecting and patching the vulnerabilities, using epidemic modeling to control and maintain green worm reproduction.

Similar methods have been previously explored by our group, which extend the Botnet Defense System (BDS) to eliminate botnet infection using white-hat worms. While the immunization strategy is effective, it requires a longer time step to eliminate infections, in addition to resources to maintain the antibody population [11]. Additionally, to stop the infection from spreading, the devices must be connected in a certain way to be effective [12]. Lastly, other research group highlight that using worms requires centralized control and infection lifespan to avoid uncontrolled propagation [13].

Machine learning (ML) tackles these issues by strategically deploying the white-hat worms to eliminate the botnets without relying on antibodies, previous white-hat worm populations, or network topology. However, supervised learning (SL)-based BDS [14] requires a balanced dataset with predefined strategies to be effective [15]. In addition, because it only supports a single bulk deployment of white-hat worms, it cannot adapt to changes in botnet infections. While reinforcement learning (RL)-based BDS [16] addressed the dataset quality and adapted to the botnet infection by launching the white-hat worms based on the infection progression, it required more training episodes to be effective.

To strengthen the RL, this article proposed the survey–identify–reinforce–optimize (SIRO) framework to reduce the number of training episodes needed for the agents to be effective. In comparison with existing research summarized in Table 1, the framework acts as a guide to implement RL workflows for eliminating botnets based on four key principles:

1. Survey: Surveyed network vulnerabilities, infection state, and changes must be encoded as a multi-channel tensor to help the agent monitor the situation;
2. Identify: Irrelevant actions must be identified and masked while allowing leniency for the agent to explore new extermination strategies;
3. Reinforce: Key actions that contribute to the agent winning must be reinforced with rewards and penalties to teach the agent;
4. Optimize: Rewards and penalties must be adjusted to consistently guide the agent to the ideal win condition.

The main contribution is the implementation of a Chebyshev-based masking strategy used in the Identify component to reduce the complexity. Consistent with our ablation results, identify contributes the largest performance gain, while Survey provides benefits primarily in combination with other components due to added complexity. To further demonstrate the generality of our approach, the framework is tested on vanilla policy gradient (VPG), deep Q-network (DQN), proximal policy optimization (PPO), and MuZero to highlight its effectiveness [17]. Compared to our previous works [16], which relies on single-channel input, illegal-move masking, and suboptimal rewards, SIRO systematically redesigns observation, action, reward, and tuning to improve the learning curve.

With the introduction concluded in Section 1, Section 2 provides the preliminaries. Then, Section 3 defines the simulation and SIRO framework implementation. The results are presented in Section 4, with the generability of this framework discussed in Section 5. Finally, Section 6 provides the conclusion and describes future works.

## 2. Preliminary

### 2.1. Grid Topology Network

**Figure 1:**
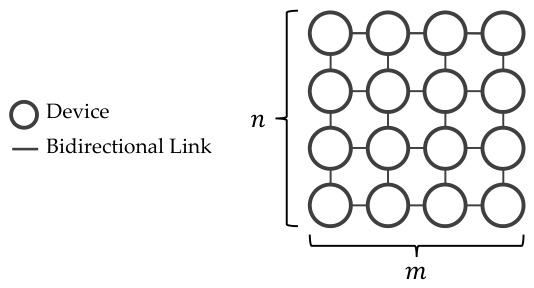
A grid topology network is defined as a network in which devices are positioned at the vertices, and the connections between neighboring vertices are bidirectional links.

A square grid topology network is a finite square lattice L(m,n) where the edges are parallel to the x-axis and y-axis [18], in addition to m=n. Each of the vertices represents the device location, and the edge between the neighboring vertices is a potential communication link. Figure 1 is an example of a grid topology network.

In this topology, two vertices are adjacent if and only if they differ by 1 in exactly one coordinate [19], which excludes any diagonal link.

### 2.2. White-Hat Worm

**Figure 2:**
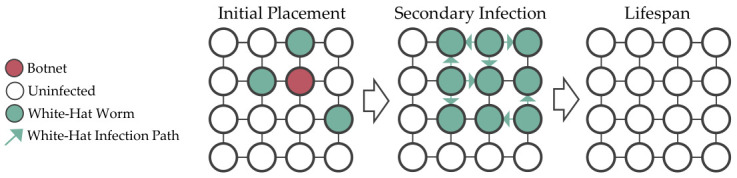
A white-hat worm is defined by its ability to cause secondary infections that reinfect and eliminate botnets from devices, and it also has a lifespan that allows it to delete itself.

A white-hat worm is a type of worm designed to combat botnet infections, aiming to safeguard the devices that are not feasible to be managed manually. The key differences that separate it from typical botnets are secondary infectivity to remove the botnet from the compromised devices and a built-in lifespan to delete itself. Figure 2 shows the white-hat worm behaviors in a 4×4 grid topology network.

### 2.3. Reinforcement Learning Algorithms

VPG is a model-free and policy-gradient method that uses Monte Carlo returns to optimize a stochastic policy directly [20]. By default, it lacks value function learning and planning, which contributes to high variance. DQN is a model-free and value-based method that uses temporal-difference updates to learn action values [21]. The training stability comes from the experience replay and target networks. PPO is a model-free and actor–critic method that combines the actor as the policy and the value estimator as the critic [22]. It uses a clipped objective to bound the per-update policy changes to improve training stability while retaining simplicity.

MuZero is a model-based method that learns the representation, dynamic, and reward by itself [23]. It achieves strong performance via off-policy training using a replay buffer. While VPG, DQN, and PPO require no planning, MuZero uses Monte Carlo Tree Search to plan. Table 2 summarizes the differences between each RL used in this article.

These algorithms are chosen because they are from four commonly used techniques in RL, which are policy-based, value-based, actor–critic, and model-based planning.

## 3. Implementation

**Figure 3:**
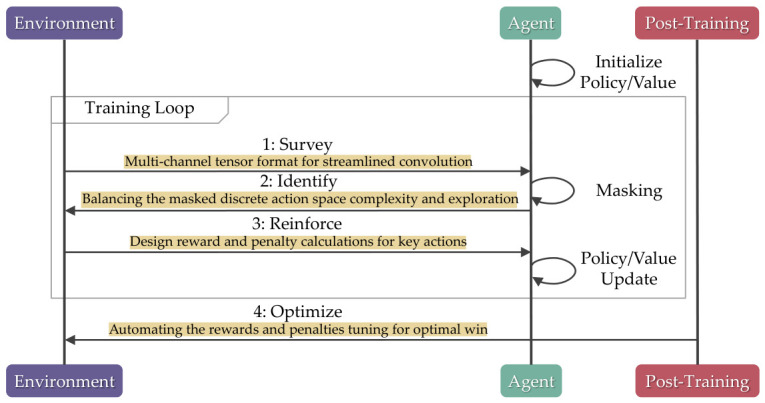
The framework augments the typical reinforcement learning workflow to ensure wider compatibility across different algorithms.

Figure 3 shows the typical RL workflow, which involves observing the environment state (Survey), prioritizing certain actions (Identify), rewarding and penalizing key actions (Reinforce), and tuning the hyperparameters (Optimize) [24]. The SIRO framework focuses on enhancing the workflow to allow adaptability to different algorithms in solving botnet infection rather than focusing on the specific policies or values of an algorithm.

The following subsections discuss the grid topology network simulation, in addition to how the SIRO framework assists the agent in understanding the network state, masking unimportant actions, reinforcing key actions with rewards, and fine-tuning the rewards to win the simulation.

### 3.1. Grid Simulation Implementation

**Figure 4:**
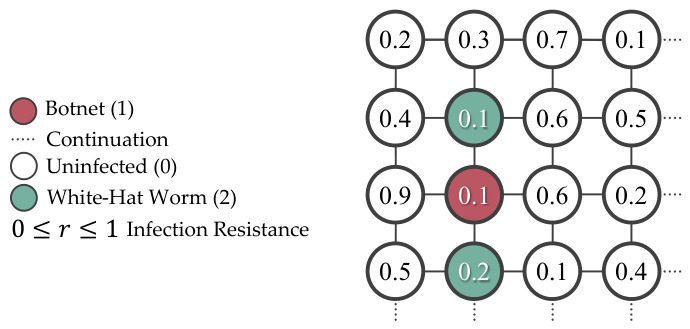
The simulation expresses the infection resistance as a decimal between 0 and 1, with uninfected devices represented as 0, botnets as 1, and white-hat worms as 2.

**Figure 5:**
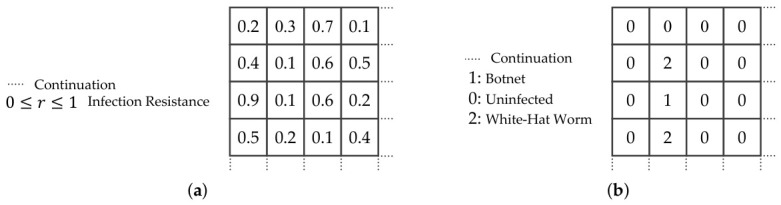
(**a**) A 2D array is used to represent the infection resiliency of each device. (**b**) Another 2D array is used to represent the infection state of each device.

The simulated network and infection model are simplified and deterministic, where the infection state is s∈{0,1,2}, the device resistance is defined as 0≤r≤1, and comparison operator decide if the botnet’s infectability exceeds the device resistance, as shown in Figure 4. To avoid overlapped numerical representation in the simulation, these values are expressed in separate 2D arrays, as shown in Figure 5.

**Figure 6:**
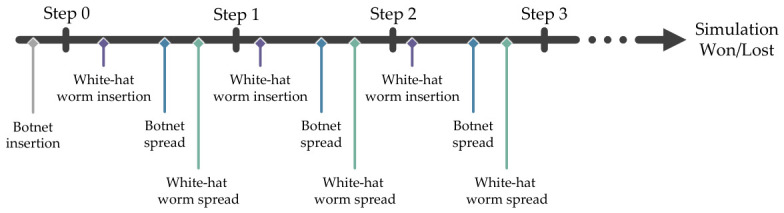
The insertion and infection spread in the simulation are predetermined to maintain determinism for debugging and evaluation. The ellipsis indicates continuation.

In the simulation, botnet insertion happen once at step 0. For the subsequent steps, the simulation will insert the white-hat worm, simulate the botnet spread, and simulate the white-hat worm spread in a predetermined sequence, which is useful for debugging and evaluating the agent’s behaviors. Figure 6 shows the simulation timeline of the grid topology network.

The botnet and white-hat worm infections spread with a Manhattan distance [25] of 1, where they infect all neighboring connected devices. While the propagation follows the same concept as other infection models [26,27,28,29], the infection model implemented in this article is simplified to infect neighboring devices where r<0.5. The stylized implementation is to speed up the simulation and shorten the time taken to train RL.

**Figure 7:**
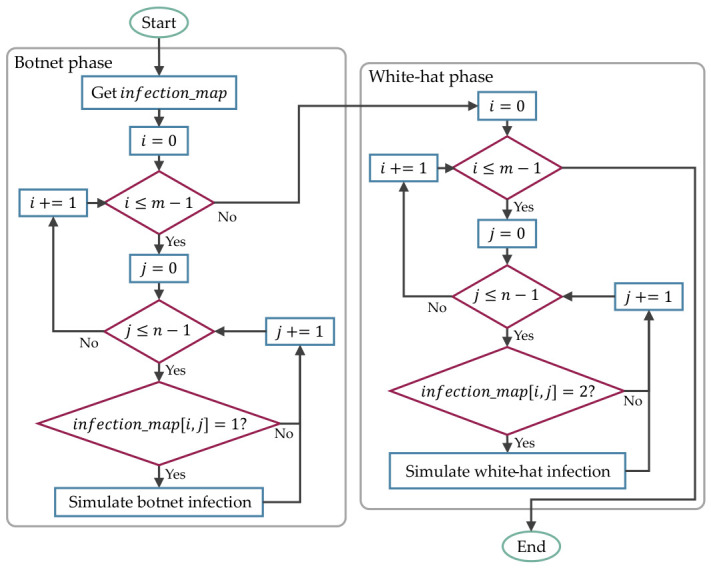
The process for simulating the botnets’ and white-hat worms’ infection in a grid topology network for every timestep.

Figure 7 shows the process for simulating the botnets’ and white-hat worms’ infection, with resilience_map and infection_map denoting the 2D arrays of device resiliency and infection state maps. The detailed implementation is defined in Appendix A, which includes the infection simulation in Algorithm A2 and the Manhattan distance of 1 in Algorithm A2. These algorithms only simulate the propagation of botnets and white-hat worms on a 2D array, with no malicious code included to adhere to the EC-Council Code of Ethics [30].

The terminal state for the simulation is shown in Equation (1), where the agent won if all botnets are removed or contained. Conversely, it loses if any botnet reaches a boundary device or the total steps taken exceed the maximum step, where total_step>max_step.

(1) $$\begin{array}{c} \mathrm{Terminal}\;\mathrm{state}=\left\{\begin{array}{cc} Win, & \mathrm{if}\;\mathrm{all}\;\mathrm{botnets}\;\mathrm{are}\;\mathrm{removed}\;\mathrm{or}\;\mathrm{contained}\\ Lose, & \mathrm{if}\;a\;\mathrm{botnet}\;\mathrm{reaches}\;\mathrm{the}\;\mathrm{boundary}\;\mathrm{or}\;total\_step>max\_step \end{array}\right. \end{array}$$

**Figure 8:**
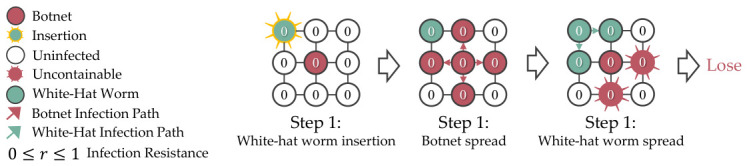
Unresolvable simulation with the botnet starting one step from the grid boundary.

**Figure 9:**
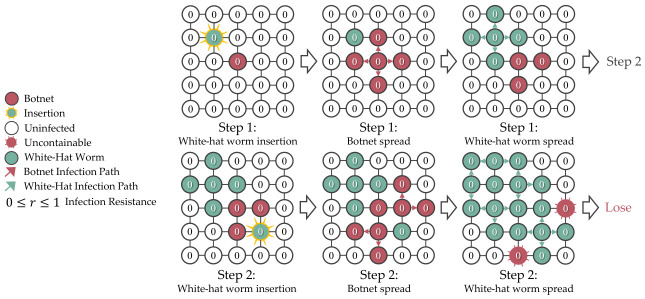
Unresolvable simulation with the botnet starting two steps from the grid boundary.

**Figure 10:**
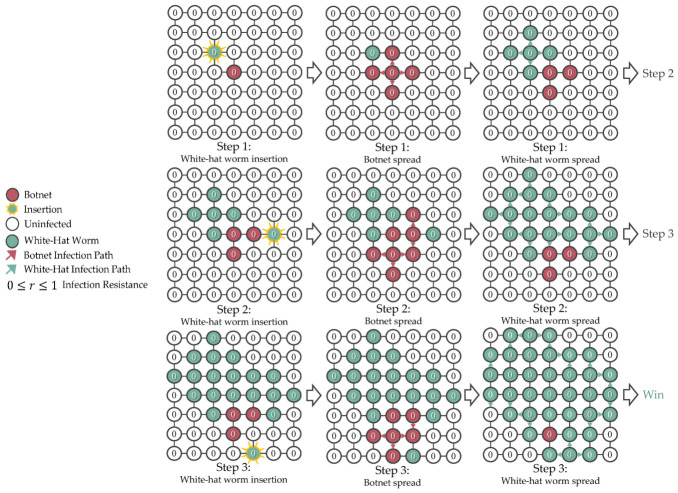
Resolvable simulation with the botnet starting three steps from the grid boundary.

**Figure 11:**
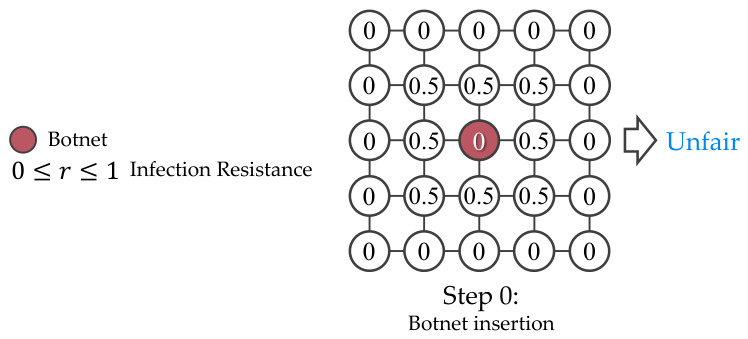
Unfair simulation where botnet was surrounded by uninfectable devices (r=0.5) at step 0.

The initial botnet placement and the infection resiliency of each device are randomly created using unique seeds for every episode to prevent overfitting and allow the agent to develop various winning strategies. To avoid unresolvable and unfair simulations, the simulation enforces a minimum grid size solvable by the RL agents and skips training on unfair networks. Figure 8, Figure 9, Figure 10 and Figure 11 are examples of unresolvable, resolvable, and unfair simulations.

To ensure the white-hat worm can catch up to the botnet, the width and height of the grid must be more than 7, as shown in Equation (2). In addition, the initial botnet insertion coordinate from the nearest coordinate must have at least a Manhattan distance of 3. With the initial botnet coordinate as initial=(i,j), where 1≤i≤m and 1≤j≤n, the minimum distance from the nearest boundary is shown in Equation (3).

(2) $$\begin{array}{c} \min(m,n)\geq 7 \end{array}$$

(3) $$\begin{array}{c} \min(i-1,m-i,j-1,n-j)\geq 3 \end{array}$$

To ensure fairness, the initial must be able to reach at least one traversable boundary device. With $\partial L_{T}$ denoting the set of traversable boundary nodes, let *b* be an element of this set. We define ${\mathrm{Reach}}_{G_{T}}\left(initial\_botnet,b\right)$ to be true if and only if there exists a path in $G_{T}$ consisting only of traversable nodes from initial to *b*, as shown in Equation (4).

(4) $$\exists \;b\in \partial L_{T}:{\mathrm{Reach}}_{G_{T}}\left(initial,b\right)$$

The simulation was implemented using Python 3.13.5 and NumPy 2.1.2 [31], where the numerical values to represent the infection state and the device’s resistance toward the infection are represented as Float 32 in NumPy’s 2D arrays. The rationale for using NumPy is to skip the step required to convert the data type to be compatible for the agent to process, shortening the time taken between each step.

### 3.2. Surveying the Situation

In RL, the agent must be able to observe and discern the situation, such as infection resistance and its state. Additionally, it will be advantageous for the agent to be able to see the past infection state and understand how the botnet infection spread.

**Figure 12:**
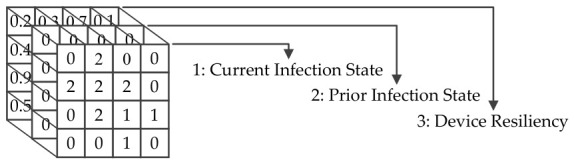
A multi-channel tensor is used to represent the current infection state, the prior infection state, and the infection resiliency of each device.

With timestep as *t*, let $infection\_map_{t-1}$ and $infection\_map_{t}$ be the grid topological network state in the previous and current timesteps. Then, the framework defined the input for the agent in Equation (5), with multi-channel tensor visualization in Figure 12.

(5) $$input=(resiliency\_map,infection\_map_{t-1},infection\_map_{t})$$

This technique is similar to stacking multiple past board states found in AlphaZero [32], with few caveats. Firstly, the framework to help the agent discern the situation does not stack the replay buffer or Markov property enforcement, which means it only provides pairs of consecutive network states compiled at times t−1 and *t*. The rationale for this approach is that the illegal move checks in a grid topology network depend solely on the current board state, which differs from chess and other board games. Secondly, instead of treating the $resiliency\_map_{t-1}$ and $resiliency\_map_{t}$ as a different set of data, they are stacked along with the board states for simplicity.

### 3.3. Identify Irrelevant Actions

Let the discrete action space be denoted as A, with the total number of actions in the discrete action space as *N*. In a grid topology network, the discrete action space can be described in Equation (6). However, the number of moves for the agent will constantly change, depending on the number of legal moves remaining and its priorities.

(6) A={1,2,…,N},whereN=n·m

**Figure 13:**
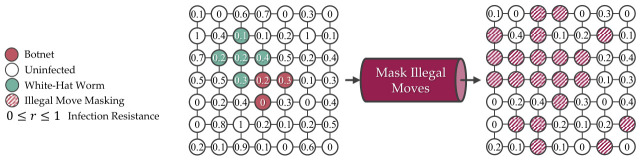
Using a comparison operator to mask illegal moves reduces the complexity in discrete action space on the L(7,7) grid.

A simple mask can be created to tell the agent to avoid inserting the white-hat worm into certain devices that it cannot infect, where the infection resiliency is r≥0.5, and if the devices are not empty, as shown in Figure 13. While it reduced the complexity in discrete action space, where the total number of actions in discrete action space is $N_{illegal}\leq N$, it does not consider the distance from the infected devices. Hence, more training episodes are needed for the agent to identify the optimal placements on the L(7,7) grid.

**Figure 14:**
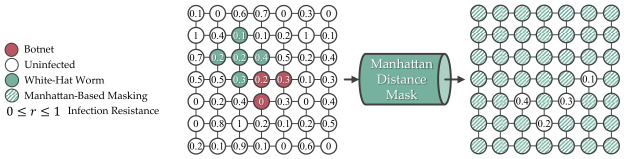
Using a Manhattan-based masking strategy further reduces the complexity, at the cost of preventing the agent from exploring different winning strategies on the L(7,7) grid.

**Figure 15:**
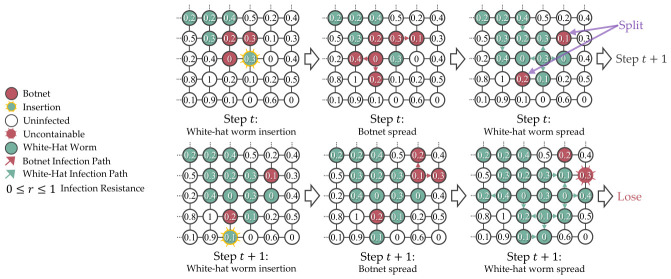
The white-hat worm placed using the Manhattan-based masking strategy could cause the botnet infection trajectory to split on the L(7,7) grid, which makes containment impossible.

To reduce the number of actions, a masking that uses a Manhattan distance of 1 from the infected devices to the infectable neighbors further reduced the complexity in discrete action space, where $N_{manhattan}\leq N_{illegal}$, as shown in Figure 14. However, such a masking will hinder the agents from exploring different strategies to contain and eliminate the botnets. Figure 15 shows a scenario where agents lost due to Manhattan-based masking prevents them from diagonally inserting the white-hat worm, which causes the botnet infection to split into two uncontainable paths on the L(7,7) grid.

**Figure 16:**
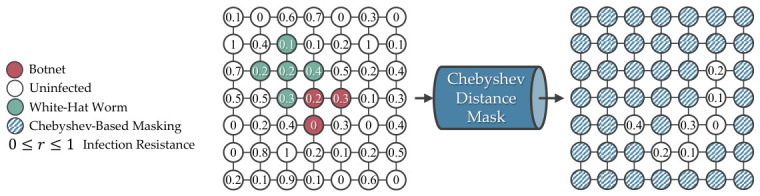
Using a Chebyshev distance of 1 to detect infectable neighboring devices slightly reduces the complexity, allowing the agent to explore different winning strategies on L(7,7).

**Figure 17:**
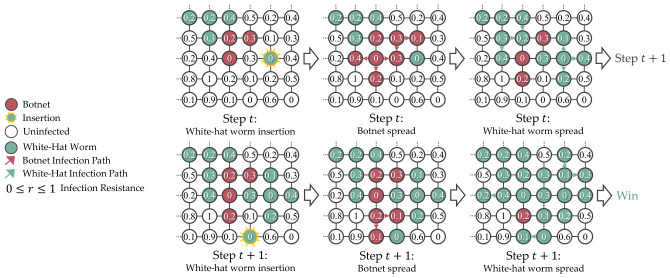
The white-hat worm placed using a Chebyshev-based masking strategy could block multiple paths when the white-hat worm spread, effectively containing the botnets.

To avoid unresolvable simulation, the framework uses a Chebyshev distance of 1 from the infected devices to the infectable neighbors to help the agent recognize important actions while allowing different winning strategies to be explored. While $N_{manhattan}\leq N_{chebyshev}$, it allows the white-hat worm to be inserted vertically, horizontally, and diagonally from the botnets, as shown in Figure 16. Figure 17 serves as another example where the agent won due to strategic diagonal insertion of the white-hat worms, similar to Figure 10.

**Figure 18:**
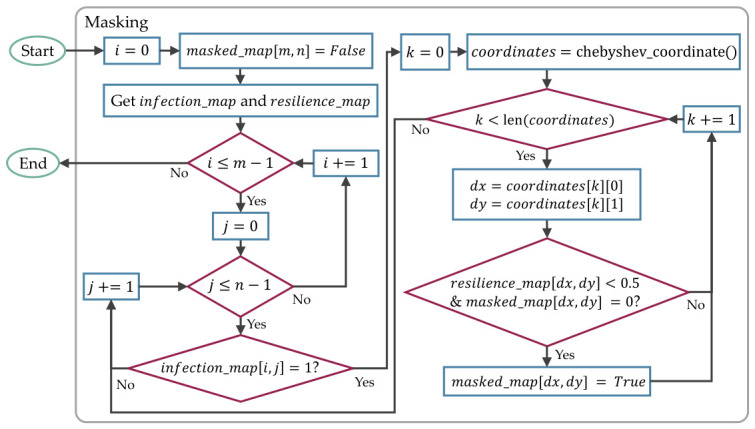
The process for masking the devices relies on a Chebyshev-based masking strategy to identify infection paths that need to be blocked.

Figure 18 is the masking process implemented to guide the agent, where a Chebyshev distance of 1 is used to find neighboring infectable devices. The detailed implementation is defined in Appendix A, which includes the masking process in Algorithm A3 and the chebyshev_coordinate () function in Algorithm A4.

A Manhattan distance of 1 directs the agent to position the white-hat worm either vertically or horizontally relative to the infected devices, while a Chebyshev distance of 1 directs the agent to place the white-hat worm either vertically, horizontally, or diagonally. In a scenario where the infection path could split off, the diagonal placement of the white-hat worm obstructs two infection pathways in a single move, avoiding the branching paths issue. For this reason, the framework uses a Chebyshev distance of 1.

### 3.4. Reinforce Agent Decisions

While masking helps the agent focus on important actions to take, it does not indicate how much those actions contribute to winning. Hence, it is necessary to establish rewards or penalties for specific actions. In RL, rewards and penalties can be classified into two types: episodic reward and step-based reward [33].

Episodic reward is often used for games or simulations with a terminal condition to let the agent know if it won or lost. Based on the terminal state in Equation (1), the winning reward win_reward and losing penalty lose_penalty are defined as constant values. Additionally, to force the agent to win with a lesser number of turns, an additional winning reward is used, where the action_efficiency reward decreases as the total_step to win increases. The framework for the episodic reward is defined in Equation (7).

(7) $$terminal=\left\{\begin{array}{c} \begin{array}{cc} \mathrm{win} & =win\_reward+action\_efficiency\left(1-\frac{total\_turn}{max\_turn}\right)\\ \mathrm{lose} & =lose\_penalty \end{array} \end{array}\right.$$

While setting up the episodic reward is straightforward, the step-based rewards are dynamically calculated based on how the network’s state changes with each step. Furthermore, while episodic rewards focus on teaching the agent how to win the game, the step-based reward teaches the agent how it should behave throughout the episode, making it possible to win with optimized actions.

To ensure the white-hat worm does not spread more than it should and to force the agent to win the game as soon as possible, the agent is penalized as the total white-hat worm count increases. With presence_scale as a constant value, $whitehat\_count_{t}$ as the total white-hat worm count on the current step, and $whitehat\_count_{t-1}$ as the total white-hat worm count on the previous step, the framework to penalize as the total white-hat worm count increases is shown in Equation (8).

(8) $$presence=presence\_scale\left(whitehat\_count_{t}-whitehat\_count_{t-1}\right)$$

To teach the agent that the actions it takes succeed in reducing the botnet infection, the agent is rewarded if it manages to reduce the botnet count from the previous step. Conversely, the agent is penalized if the botnet count increases from the previous step. With progress_scale as a constant value, $botnet\_count_{t}$ as the total botnet count on the current step, and $botnet\_count_{t-1}$ as the total botnet count on the previous step, the framework to reward or penalize as the total botnet count changes is shown in Equation (9).

(9) $$progress=progress\_scale\left(botnet\_count_{t-1}-botnet\_count_{t}\right)$$

To tell the agent if it is successful in controlling the botnet’s infection, the reward is given only if white-hat worms block the shortest botnet infection path to the boundary devices, with bonus rewards given for additional blocked paths. With block_scale as a constant value, bfs_boundary() as the helper function to find the shortest distance to the boundary devices using breadth-first search, and group_helper() as a helper function to classify the measured distance, the framework to reward the white-hat worms that block infection paths is shown in Algorithm 1.
**Algorithm 1** Calculate the rewards for blocking the botnet infection paths.**Input:** Current mask $masked\_map_{t}$, previous mask $masked\_map_{t-1}$, resilience_map**Output:** Blocked path rewards block  1:blocking_rewards$\left(masked\_map_{t},masked\_map_{t-1},resilience\_map\right)$  2:   Initialize the reward scale: block_scale  3:   Initialize a list to store the results: results←[]  4:   Initialize an integer to count blocked paths: paths←0  5:   Initialize a dictionary to group the results: groups←{‘shortest’:[],‘else’:[]}  6:   Find *t* unmasked coordinates: current_indices←find_true ($masked\_map_{t}$)  7:   Find t−1 unmasked coordinates: previous_indices←find_true ($masked\_map_{t-1}$)  8:   **for** x,y **in** previous_indices **do**  9:      Find the shortest distance to boundary: distance←bfs_boundary (x,y)10:      Save the results: results.append ([(x,y),distance])11:   **end for**12:   Group the results based on distance: groups←group_helper (results)13:   Find missing coordinates: missing←set (previous_indices)−set (current_indices)14:   Convert set into a list: missing←list (missing)15:   **for** coordinate,distance **in** groups[‘shortest’] **do**16:      **if** coordinate **in** missing **do**17:         Count the blocked paths: paths←paths+118:   **end for**19:   **if** paths>0 **do**20:      **for** coordinate,distance **in** groups[‘else’] **do**21:         **if** coordinate **in** missing **do**22:            Count the blocked paths: paths←paths+123:   **end for**24:   Calculate the rewards: block←block_scale·paths25:   **return** block26:**End Function**

The total reward given at the end of each step is shown in Equation (10), where the episodic reward is only given when the simulation reaches its terminal state. Furthermore, the hand-tuned hyperparameters for the rewards are defined in Table 3.

(10) $$total\_reward=presence+progress+block+\left\{\begin{array}{cc} terminal, & \mathrm{if}\;\mathrm{terminal}\\ 0, & \mathrm{otherwise} \end{array}\right.$$

While the rewards are designed to incentivize the agent for efficiently eliminating or containing the botnet infection, there is a risk that changes in network size and device resiliency could cause the step-based rewards to overshadow the episodic rewards, leading the agent to intentionally increase the number of steps taken to accumulate more step-based rewards.

### 3.5. Optimize Rewards Incentives

**Figure 19:**
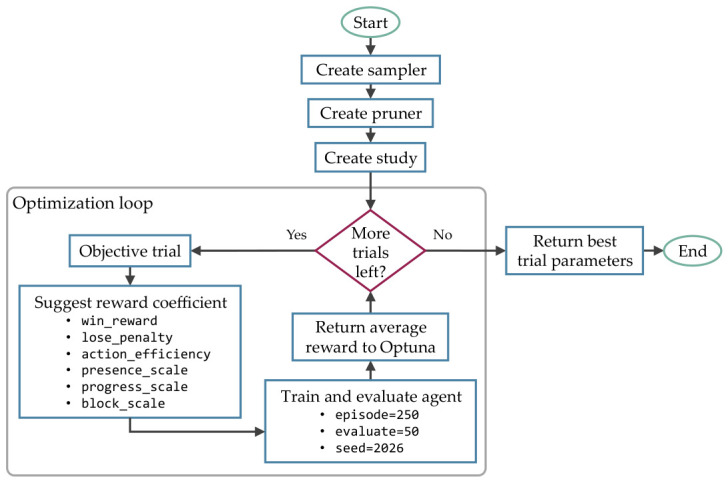
The process to optimize the reward and penalty coefficients utilized Optuna to tune the block_scale, win_reward, lose_penalty, presence_scale, progress_scale, and action_efficiency.

Although the rewards and penalties can be hand-tuned to consistently guide the agent to win with the most optimized moves, it took a long time for a human to fine-tune them for each RL algorithm. To automate the process and avoid brute-forcing the entire combinations, Optuna 4.6.0 [34] was utilized to implement Bayesian optimization, as shown in Figure 19.

Specifically, Optuna was used to implement Tree-Structured Parzen Estimator, which is a Bayesian optimization that utilizes probabilistic models as the objective function to search the hyperparameter space efficiently. With a total of 30 trials, the agent is trained with 250 episodes and evaluated with 50 episodes, where seeded randomization is used to generate networks. The value ranges for Optuna to optimize are shown in Table 4.

Once the best reward coefficients are found, the agent will undergo training and evaluation, which will be discussed further in the following section.

## 4. Evaluation

In this section, the hyperparameters used to configure the RL algorithms and the ML architectures are defined. Next, the experiments on the previous RL-based BDS launcher [16] against the SIRO framework are compared. Then, the scalability of the SIRO framework on DQN is tested. Finally, an ablation study was conducted to evaluate how the components in SIRO impact accuracy.

### 4.1. Reinforcement Learning Configuration

In this article, the RL algorithms are configured with the same hyperparameters throughout the experiments, as shown in Table 5 for VPG, Table 6 for DQN, Table 7 for PPO, and Table 8 for MuZero. These hyperparameters represent the default values used to initialize the algorithms with no fine-tuning, except for the number of simulations in MuZero, which was reduced to 10 for shorter training times.

For consistency, all RL algorithms utilized the same convolutional neural network (CNN)-based encoder model. The CNN-based encoder model is chosen because it helps the RL algorithms to capture the spatial pattern in 2D networks, where neighboring nodes influence each other. This rationale aligns with the past research done in AlphaZero to solve the board game problems [32].

**Figure 20:**
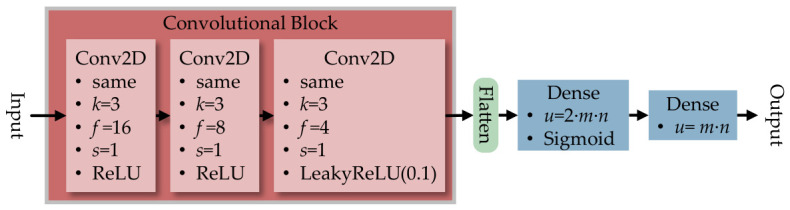
The convolutional neural network-based encoder model is used on all reinforcement learning algorithms for consistency in analyzing the grid-topology network.

It utilized three 2D convolution layers (Conv2D), with padding set to same to retain the dimension size, followed by kernel *k*, filter *f*, and stride *s* values shown in Figure 20. The rectified linear unit (ReLU) is used for the first and second Conv2D layers, while the leaky rectified linear unit (LeakyReLU) of 0.1 is used in the final layer to avoid the vanishing gradient problem. Once flattened, the data is parsed to the dense layer with the unit number of u=2·m·n to reduce the dimensionality. A sigmoid activation then converts the output into a probability-like value between 0 and 1 to place the white-hat worm before the final dense layer of u=m·n adjusts the output to match the network.

**Figure 21:**
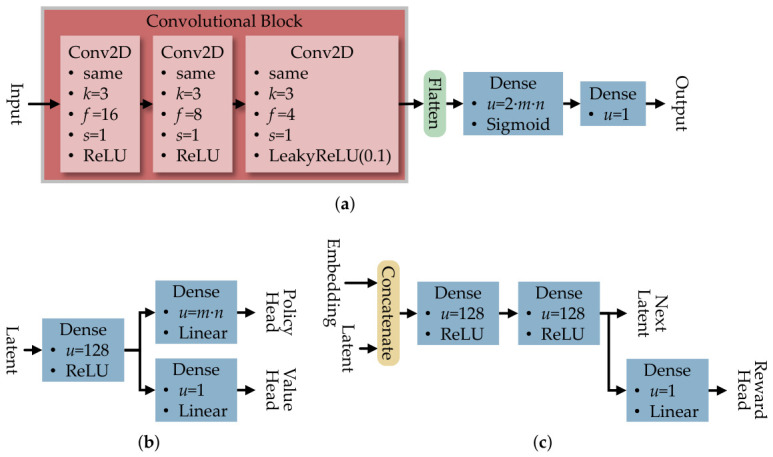
(**a**) The convolutional neural network-based encoder is used as the critic network in proximal policy optimization. (**b**) The multi-task prediction network is used to predict the next hidden state and reward in MuZero. (**c**) The multi-task dynamic network is used to estimate the policy and value of a hidden state in MuZero.

For RL algorithms that utilize multiple ML models, like PPO and MuZero, additional ML models are implemented, as shown in Figure 21. The critic network in PPO reused the CNN-based encoder model, where the output was changed into one unit in Figure 21a. Meanwhile, the prediction network and the dynamic network in MuZero are implemented with multi-task learning models shown in Figure 21b,c.

In this article, PyTorch 2.8.0 [35] is used to implement the RL algorithms. Furthermore, the hyperparameters for the algorithms were purposely not fine-tuned, and the ML models used were standardized to highlight the improvements from using the SIRO framework.

### 4.2. Performance Comparison Study

To compare the performance against the SIRO framework, the rewards and penalties designs, illegal moves masking, and single-channel tensor for representing the current infection state that are used in the previous RL-based BDS launcher [16] were implemented on VPG, DQN, PPO, and MuZero. While the illegal moves masking and current infection state tensor have been discussed in Section 3, the rewards and penalties for the previous RL setup are discussed in Appendix B, with total reward computation in Equation (A6).

The agents configured with the previous configurations from the RL-based BDS launcher and improved with the SIRO framework are trained on 1000 seeded grid topology networks with dimensions L(7,7), with the initial botnet being inserted at the center to guarantee the simulation’s solvability. The agents are evaluated at 10-episode training intervals using 100 untrained seeded episodes to determine the performance. The training and evaluation were conducted three times to obtain the mean.

**Figure 22:**
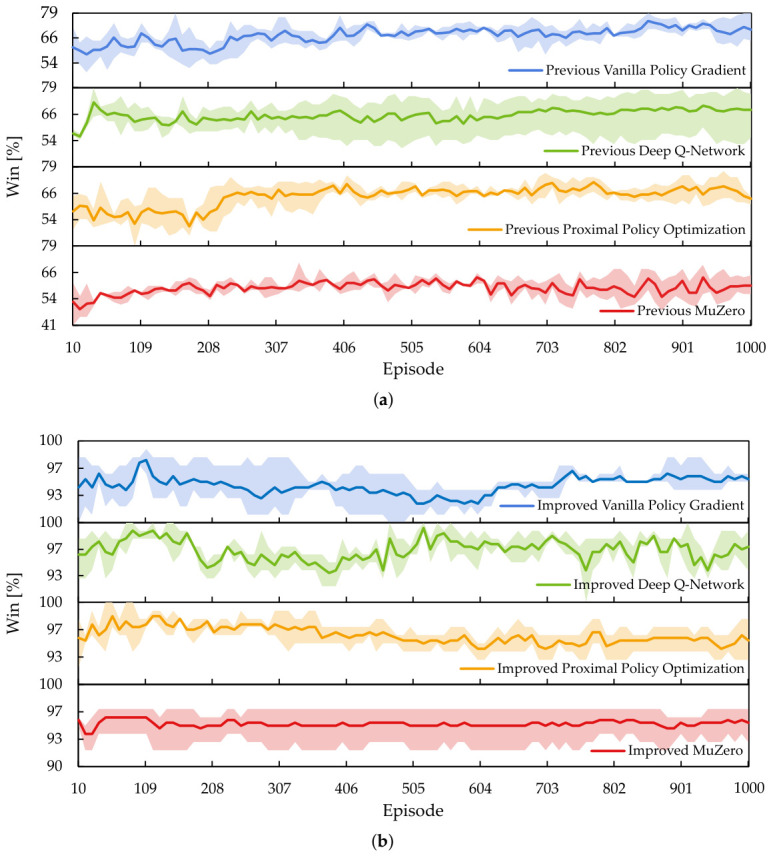
The mean line plot with minimum and maximum values as a range band on the winning rate. (**a**) Most of the agents configured with the previous reinforcement learning setup improve slowly and plateau below a 80% winning rate. (**b**) The agents improved with the survey–identify–reinforce–optimize framework exceeded 90%, with some achieving a 100% winning rate.

Figure 22 is the winning rate score, with a range band showing the minimum and maximum values observed during the evaluations. In Figure 22a, some of the agents configured with the previous RL setup slowly improve as the number of training episodes increases. However, the winning rate mostly plateaued for these agents, and they struggled to exceed 85%. In contrast, all agents improved with the SIRO framework in Figure 22b exceeding 90%, with DQN and PPO successfully achieving a 100% winning rate in shorter training episodes.

The results show that agents that used the previous RL setup could not learn winning strategies within 1000 training episodes because of the sheer complexity of the discrete action space in the grid topology network. Conversely, the Chebyshev-based masking strategy used in the SIRO framework helped the agents focus on important actions, while rewards and penalties optimized using Optuna encourage the agents to make decisions that increase their chances of winning.

**Figure A1:**
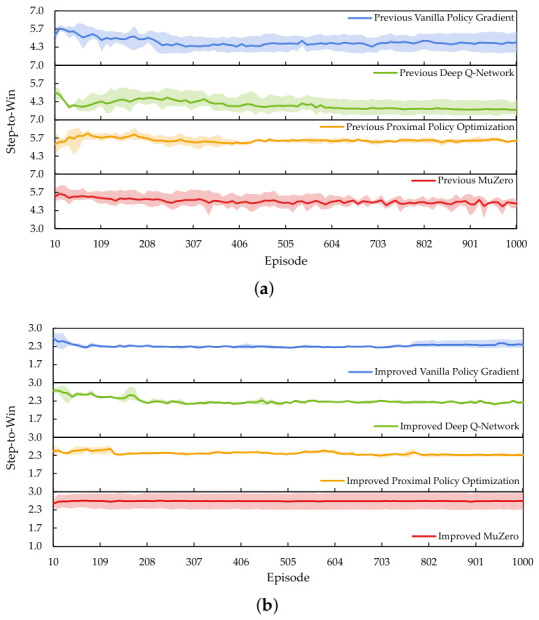
The mean line plot with range band on step-to-win. (**a**) The agents configured with previous reinforcement learning setup slightly lowered the step-to-win before plateauing above three steps. (**b**) The agents configured with the survey–identify–reinforce–optimize quickly reduced and maintained the step-to-win under three steps.

In addition to this, the step taken to win the game, aptly named “step-to-win,” is also measured and visualized using a line plot with a range band in Appendix C, Figure A1, with detailed analysis in Table A1. While agents implemented with the SIRO framework are faster in winning the game, the step-to-win metric does not take into account the simulations the agents lost. With 1 as the smallest possible step to win and max_step=10, the step-to-win can be expressed as win efficiency using Equation (11).

(11) $$win\_efficiency=winning\_rate\cdot \frac{10-step\_to\_win}{10-1}$$

**Figure 23:**
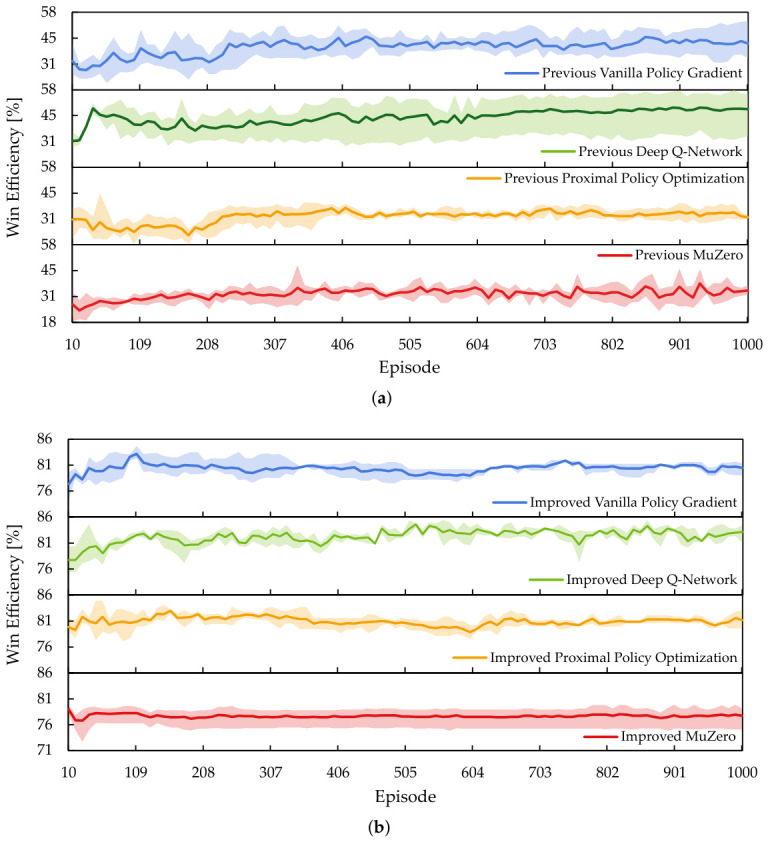
The mean line plot with minimum and maximum values as a range band on the win efficiency. (**a**) The agents that are configured using the previous reinforcement learning setup struggle to improve their win efficiency beyond 60%. (**b**) The agents improved with the survey–identify–reinforce–optimize framework, maintaining a mean win efficiency above 70%.

Figure 23 is the mean win efficiency score, with minimum and maximum values observed during the evaluations shown as the range band. In Figure 23a, the agents configured with the previous RL setup struggle to improve their win efficiency beyond 60%. However, the agents improved with the SIRO framework in Figure 23b and exceeded and maintained win efficiency above 70%. The results indicate that SIRO agents win faster and more efficiently.

While Figure 22 and Figure 23 are intuitive for visualizing the mean, minimum, and maximum values, it is difficult to numerically compare the standard deviation between the agents configured using the previous RL setup and the SIRO framework. To solve this, the normalized area under the learning curve (AULC) [36] for each run was calculated before using it to obtain the standard deviation.

Since the agents evaluated at 10-episode training intervals yield 100 results, with *y* representing the measured performance, the AULC can be calculated using the trapezoidal rule in Equation (12). Then, with evaluation starting from the tenth episode and each evaluation containing 100 untrained episodes, the normalized AULC can be calculated using Equation (13). Finally, the standard deviation was calculated from the normalized AULCs.

(12) $$\begin{array}{c} \mathrm{AULC}=\sum_{episode=1}^{100-1}\frac{y_{episode}+y_{episode+1}}{2}\cdot 10 \end{array}$$

(13) $$\begin{array}{c} \mathrm{Normalized}\;\mathrm{AULC}=\frac{\mathrm{AULC}}{100\left(1000-10\right)} \end{array}$$

Table 9 is the normalized mean AULC with standard deviation. The results confirmed the observations in Figure 22, showing that the agents who improved with the SIRO framework had mean and normalized AULC values closer to 1, indicating ideal learning behavior. Additionally, most agents that were improved with the SIRO framework exhibit lower standard deviation values.

Similarly, Table 10 shows that agents who improved using the SIRO framework had normalized mean AULC values closer to 1 and exhibited lower deviation. The results show that the SIRO framework is much more efficient in winning the game with fewer steps, which confirms the observation in Figure 23 and Figure A1.

Finally, to obtain the mean improvement between the previous RL setup and the SIRO framework, the mean percentage change for each agent was computed and shown in Table 11. The results show that the mean SIRO framework improvement across the RL algorithms on the winning rate is 49.1293% and the win efficiency is 118.8031%, confirming the effectiveness of the SIRO framework in improving the agents.

### 4.3. Network Scalability Study

To test the scalability of the SIRO framework, a DQN-based agent was chosen due to having less computational complexity while having good performance compared to other algorithms. The DQN-based agent is tested on grid topology network sizes from L(7,7) to L(28,28), with the ML model in Figure 20 scaled to the network size.

The training and evaluation using the same parameters in Section 4.2 were repeated three times, where the normalized AULCs were measured and the mean and standard deviation were computed. In this test, the initial botnet was inserted at the center for L(7,7). For L(8,8) onward, it was randomized while adhering to the requirement in Equation (3).

**Figure 24:**
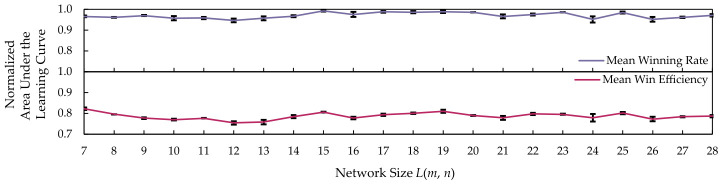
The plotted area under the learning curves for the deep Q-network agent shows slight improvement as the network size increases, before oscillating on L(21,21) onward.

Table 12 is the computed mean and standard deviation for the DQN-based agents, in addition to the line plot with error bars shown in Figure 24. The winning rate slightly increases from L(15,15) to L(20,20) before oscillating at L(21,21) onward. Similar patterns are observed in win efficiency. Both metrics display a small standard deviation.

The winning rate for DQN with the SIRO framework increases as the network size grows because larger grids provide more opportunities for DQN to place the white-hat worms, which help eliminate the botnet and prevent it from reaching the boundary devices. The oscillation of the area under the learning curves on L(21,21) is due to a limitation of the ML model in DQN itself, not the SIRO framework. Although the input and output of the ML model are designed to dynamically adjust to the network size, the number of layers remains constant, which could hinder DQN with the SIRO framework from scaling up.

### 4.4. Reward Ablation Study

To evaluate the contribution of each of the components and their combinations in SIRO framework, an ablation study was conducted using DQN-based agents on an L(7,7) grid network by implementing individual and different combinations of the components. Then, the training and evaluation using the same parameters in Section 4.2 were repeated three times, where the normalized AULCs were measured.

Table 13 shows the mean and standard deviations for the normalized AULCs on each of the components and their combinations. The result on DQN utilizing only the Survey component has the lowest mean normalized AULC, which is lower than the previous RL setup. The reason for this result is that using multi-channel tensor input increases complexity without support from other components. Conversely, the identify component provides the highest mean AULC improvements, highlighting the advantage of using a Chebyshev-based masking strategy.

**Figure 25:**
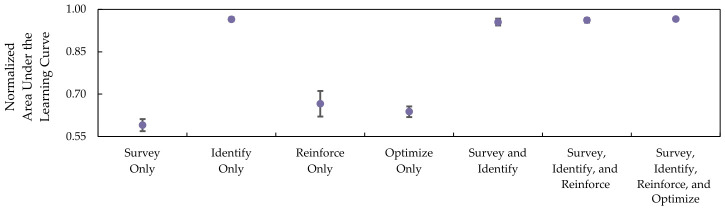
The winning rate is represented by the mean and normalized area under the learning curves, with error bars indicating the standard deviation of each component.

**Figure 26:**
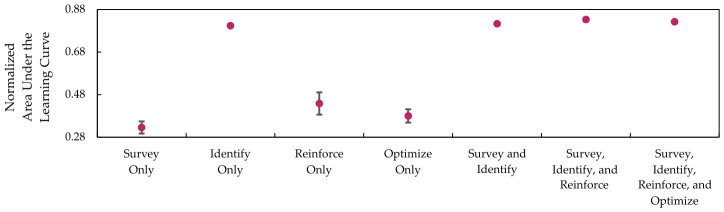
The win efficiency is represented by the mean and normalized area under the learning curves, with error bars indicating the standard deviation of each component.

The differences in AULC scores from combining multiple components from the SIRO framework are less noticeable. Hence, Figure 25 and Figure 26, which represent the scatter plot with error bars, are used to visualize the winning rate and win efficiency.

Both figures show that combining the components slightly improves the winning rate and win efficiency, with the exception of a complete SIRO framework implementation. While it has the highest mean AULC on winning rate, the mean AULC on win efficiency is slightly lower when compared to the DQN-based agent implemented with Survey, Identify, and Reinforce components. The reason for this result is that Optuna optimizes the scale to achieve a higher winning rate instead of winning efficiency. In other words, at the cost of slightly increasing the step-to-win, the DQN agent will have a higher chance to win.

In addition to the ablation study on each of the components in the SIRO framework, the scales used for rewards and penalties are also evaluated. With the same parameters where all the components in SIRO were utilized, the training and evaluation were repeated three times, and the normalized AULCs were measured.

The baseline result, which represents the conditions where all step-based rewards and penalty optimized by Optuna are enabled, followed by the results of each optimized step-based reward and penalty optimized by being disabled, is shown in Table 14.

As anticipated, the mean winning rate is higher when all step-based rewards and penalty are enabled in the baseline, followed by the individual scales of block_scale, presence_scale, action_efficiency, and progress_scale. The result shows that blocking the path has a great influence on the winning rate, followed by the rewards and penalties that force the agent to win with fewer steps. In other words, reinforcing the DQN-based agent to win as soon as possible help to reduce the chance of botnet from spreading.

**Figure 27:**
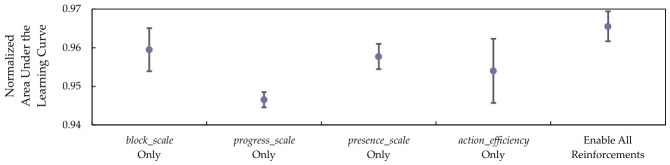
The winning rate is represented by the mean and normalized area under the learning curves, with error bars indicating the standard deviation of each reinforcement.

**Figure 28:**
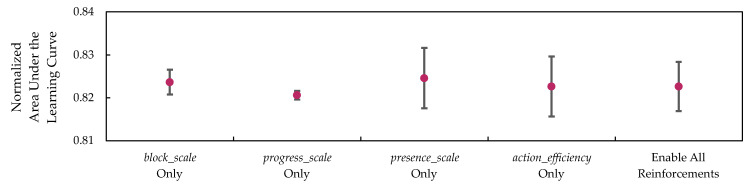
The win efficiency is represented by the mean and normalized area under the learning curves, with error bars indicating the standard deviation of each reinforcement.

To visualize the standard deviations, Figure 27 and Figure 28 represent the scatter plot with error bars for the winning rate and win efficiency. From these figures, presence_scale has the highest level of variability or inconsistency in both winning rate and win efficiency. This result occurs because the DQN-based agent is rewarded for winning as quickly as possible, which may lead it to make suboptimal decisions that only temporarily contain the botnets in the long term.

These results confirmed the contribution of each component in the SIRO framework and the step-based rewards and penalty to ensure a high winning rate and win efficiency.

## 5. Discussion

The evaluation conducted in Section 4.3 shows that the agent reinforced with the SIRO framework could be scaled up until L(20,20) before the winning rate starts to oscillate on L(21,21) onward. However, the previous evaluation has only been done from L(7,7) to L(28,28), which poses new questions about scaling beyond L(28,28) and when m≠n.

**Figure 29:**
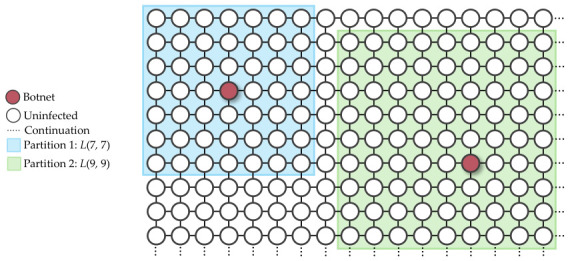
To scale beyond the grid topology network it was not trained with and m≠n, grid partitioning is recommended for generalization.

To answer these, as long as the network’s grid topology meets the requirement outlined in Equation (2), it can be partitioned into smaller grids, provided that no partition contains two infected devices. Additionally, each infected device is at least three steps away from the new boundary, as outlined in Equation (3) and shown in Figure 29.

While this article focuses on the grid topology network due to ease of interpretation, simulation, and implementation, scale-free networks are more commonly used, especially in IoT environments [37,38,39]. To use the SIRO framework on the scale-free networks, the ML model must learn the network connection, in addition to adapting the Chebyshev distance mask to the scale-free network.

To capture the spatial pattern in a scale-free network, the ML model employed by the agent should use a graph neural network (GNN) to represent the connection of the scale-free network and substitute the convolutional block in Figure 20. This approach could shorten the training time needed because the edges and vertices inside the GNN already represent the network connection, eliminating the need for the agent to painstakingly learn the connection with more training episodes.

However, this technique assumes that the links between the devices in the scale-free network are fixed throughout the training and evaluation. If the connection between the devices changes, the edges inside the GNN must be updated and retrained for it to be effective in dealing with the botnet threat in a scale-free network. This limitation also prevents the partitioning technique for handling larger scale-free networks from being effective, as the time taken to update and retrain the model is long enough for the botnet infection to spread.

To emulate the Chebyshev distance behavior for finding vertically, horizontally, or diagonally neighboring devices, a breadth-first search (BFS) algorithm could be used to anticipate the botnet spread in a scale-free network [40]. Algorithm 2 is the Chebyshev distance of 1 emulation using BFS on a scale-free network.
**Algorithm 2** Chebyshev distance of 1 emulation on a scale-free network.**Input:** Graph G=(V,E) as adjacency lists Adj[·]; source device s∈V; threshold k←2**Output:** Set $N_{\infty }\left(s\right)$: nodes considered at Chebyshev distance 1 from *s*  1:bfs_neighbors (G,s,k)  2:   Initialize empty set of 1-hop neighbors: $N_{1}\leftarrow \emptyset$  3:   Initialize empty dictionary of sets: parents[u]←∅ for all u∈V  4:   Initialize queue: Q←[]  5:   // tuple: (node, depth, first_hop), ⊥=none  6:   Enqueue (s,0,⊥) into *Q*  7:   **while** *Q* is not empty **do**  8:      (v,d,fh)←Q.dequeue()  9:      **if** d=0 **do**10:         **for each** w∈Adj[v] **do**11:            Enqueue (w,1,w) into *Q*12:         **end for**13:      **else if** d=1 **do**14:         // Find neighboring devices (vertical and horizontal)15:         $N_{1}.\mathrm{add}\left(v\right)$16:         **for each** u∈Adj[v] **do**17:            **if** u=s **then continue**18:            parents[u].add(v)19:            Enqueue (u,2,v) into *Q*20:         **end for**21:      **else if** d=2 **then**22:         **continue**23:   **end while**24:   // Find devices at distance 2 with ≥*k* distinct first-hop parents (diagonal)25:   $N_{2}\leftarrow \{\;u\in V|u\neq s\wedge u\notin N_{1}\wedge |\mathrm{parents}\left[u\right]|\geq k\;\}$26:   **return** $N_{\infty }\left(s\right)\leftarrow N_{1}\cup N_{2}$27:**End Function**

Using BFS to identify first-hop neighboring devices from an infected device retrieves all devices directly connected to it in a scale-free network. This emulates identifying devices that are connected vertically and horizontally in a grid topology using a Chebyshev distance of 1. Furthermore, BFS finds second-hop devices that are reachable from at least two distinct first-hop neighboring devices, which can be used to block two or more infection routes in the scale-free network. This behavior is analogous to identifying diagonally positioned devices in a grid topology using a Chebyshev distance of 1.

These workarounds on GNN and masking show the possibilities of adapting the SIRO framework to scale-free networks, with scalability limitations reserved for future works.

## 6. Conclusions

In this article, the SIRO framework is proposed to improve the RL-based BDS launcher to eliminate botnet infections in the stylized grid-topology network simulation. An efficient RL-based BDS launcher is achievable within 1000 episodes by following four key principles: (1) survey, (2) identify, (3) reinforce, and (4) optimize.

When compared to the previous RL setup [16], the SIRO framework successfully increases the mean winning rate by 49.1293% and improves the mean win efficiency by 118.8031% in L(7,7), with the best performers achieving 100% botnet elimination within 2.3500 steps, as shown in Table A1. Furthermore, a DQN-based agent that utilizes the SIRO framework has a higher winning rate from L(15,15) to L(20,20). The masking utilized by the SIRO framework is the primary reason for performance gains by reducing discrete action space complexity. Complementing this, the Bayesian optimization done by Optuna to fine-tune the rewards and penalties gives an additional boost in performance. This helps to avoid the step-based rewards from overshadowing the episodic rewards, leading the agent to increase the number of steps taken to accumulate more rewards.

While the article proposes the SIRO framework to address the long training time and low effectiveness of RL-based BDS launchers in a grid topology network, the same framework could be adapted to a scale-free network. By emulating the Chebyshev distance of 1 using the BFS algorithm, it is possible to identify the optimal white-hat placement that could effectively block multiple infection routes. Furthermore, by adapting the GNN as a replacement for the CNN layers, with the edges and vertices representing the connections and devices in the scale-free network, the number of training episodes required could be reduced. However, the partitioning technique that addresses the scalability issue in grid topology networks is not effective in scale-free networks.

In future work, we aim to expand the RL-based BDS launcher strategies to handle multiple botnet insertions and evaluate their performance on scale-free networks, including the use of partitioning techniques and addressing adversarial attacks [41]. Furthermore, we aim to improve the environment’s source code, provide the documentation, and release it as a Python module to make it more accessible in the future.

## Figures and Tables

**Table 1:** Existing research that requires specific conditions and high-quality datasets to be effective against the framework proposed in this article.

Authors	Device Immunity	Network Topology	Benign Worms	White-Hat Worm Launcher	
				Bulk	Timestep
Muthukumar et al. [8]	✓				
Rodríguez et al. [9]		✓			
Asadi et al. [10]	✓		✓		
Okawa et al. [11]	✓				
Tatebatake et al. [12]		✓			
Jia et al. [13]			✓		
Pan et al. [14]				✓	
Yoshioka et al. [16]					✓*
This article					✓

* It requires more training episodes to be effective.

**Table 2:** The reinforcement learning algorithms used to evaluate the effectiveness of the proposed framework.

Algorithm	Model	Data	Optimization	Planning
Vanilla policy gradient	Model-free	On-policy	Policy-gradient	No
Deep Q-network	Model-free	Off-policy	Q-learning	No
Proximal policy optimization	Model-free	On-policy	Actor-critic	No
MuZero	Model-based	Off-policy	Policy, value, and model	Yes

**Table 3:** The hand-tuned hyperparameter values for the scales are used to obtain the episodic and step-based reinforcement, with a positive sign for rewards and a negative sign for penalties.

Scale	Sign	Value	Type
block_scale	+	0.05	Reward
win_reward	+	0.5	Reward
lose_penalty	−	1	Penalty
presence_scale	−	0.005	Penalty
progress_scale	+	0.2	Reward
action_efficiency	+	0.5	Reward

**Table 4:** The reward and penalty value ranges used by Optuna to identify the optimal reward coefficient over 30 trials, with a positive sign for rewards and a negative sign for penalties.

Scale	Sign	Value Range	Type
block_scale	+	0–0.2	Reward
win_reward	+	0.1–2	Reward
lose_penalty	−	0.5–2	Penalty
presence_scale	−	0–0.05	Penalty
progress_scale	+	0–1	Reward
action_efficiency	+	0.1–1	Reward

**Table 5:** The hyperparameters used to configure the vanilla policy gradient.

Vanilla Policy Gradient’s Hyperparameters	Value
Gamma	0.99
Batch size	64
Epoch number	10
Adam’s learning rate	0.0003

**Table 6:** The hyperparameters used to configure the deep Q-network.

Deep Q-network’s Hyperparameters	Value
Epsilon	1
Gamma	0.99
Batch size	64
Memory size	100,000
Epsilon decay	0.995
Update interval	1000
Minimum epsilon	0.05
Adam’s learning rate	0.0003

**Table 7:** The hyperparameters used to configure the proximal policy optimization.

Proximal Policy Optimization’s Hyperparameters	Value
Gamma	0.99
Batch size	64
Policy clip	0.2
Epoch number	10
Adam’s learning rate	0.0003
Generalized advantage estimation	0.95

**Table 8:** The hyperparameters used to configure the MuZero.

MuZero’s Hyperparameters	Value
Gamma	0.8
Unroll steps	5
Latent dimension	128
Maximum memory	50,000
Adam’s learning rate	0.001
Number of simulation	10
Maximum episodes kept	2000
Embedded action dimension	32
Predictor-based upper confidence tree constant	1

**Table 9:** The normalized area under the learning curve for winning rate with standard deviation across different reinforcement learning agent implementations.

Algorithm	Implementation	Mean	Standard Deviation
Vanilla policy gradient	Previous	0.6765	0.0206
	Improved	0.9456	0.0129
Deep Q-network	Previous	0.6581	0.0815
	Improved	0.9655	0.0039
Proximal policy optimization	Previous	0.6476	0.0098
	Improved	0.9601	0.0041
MuZero	Previous	0.5885	0.0149
	Improved	0.9521	0.0212

**Table 10:** The normalized area under the learning curve for win efficiency with standard deviation across different reinforcement learning agent implementations.

Algorithm	Implementation	Mean	Standard Deviation
Vanilla policy gradient	Previous	0.4001	0.0547
	Improved	0.8037	0.0065
Deep Q-network	Previous	0.4386	0.0931
	Improved	0.8226	0.0057
Proximal policy optimization	Previous	0.3253	0.0069
	Improved	0.8094	0.0047
MuZero	Previous	0.3263	0.0348
	Improved	0.7764	0.0200

**Table 11:** The mean percentage change for the area under the learning curve on winning rate and win efficiency.

Algorithm	Winning Rate Improvement [%]	Win Efficiency Improvement [%]
Vanilla policy gradient	39.7780	100.8822
Deep Q-network	46.7129	87.5708
Proximal policy optimization	48.2453	148.8404
MuZero	61.7809	137.9191
Mean	49.1293	118.8031

**Table 12:** The area under the learning curves for the deep Q-network agent slightly improves as the network size increases, before oscillating on L(21,21) and onward.

Network Size L(m,n)	Winning Rate		Win Efficiency	
	Mean	Standard Deviation	Mean	Standard Deviation
7	0.9655	0.0039	0.8226	0.0057
8	0.9611	0.0016	0.7961	0.0006
9	0.9697	0.0026	0.7773	0.0045
10	0.9571	0.0106	0.7701	0.0042
11	0.9577	0.0053	0.7771	0.0011
12	0.9463	0.0082	0.7544	0.0077
13	0.9566	0.0098	0.7588	0.0107
14	0.9671	0.0048	0.7842	0.0076
15	0.9926	0.0045	0.8064	0.0025
16	0.9753	0.0115	0.7776	0.0063
17	0.9875	0.0044	0.7934	0.0055
18	0.9858	0.0052	0.8008	0.0032
19	0.9887	0.0063	0.8109	0.0074
20	0.9854	0.0012	0.7901	0.0024
21	0.9657	0.0096	0.7786	0.0093
22	0.9750	0.0048	0.7978	0.0052
23	0.9858	0.0009	0.7957	0.0029
24	0.9514	0.0148	0.7797	0.0180
25	0.9835	0.0047	0.8019	0.0057
26	0.9517	0.0120	0.7730	0.0110
27	0.9608	0.0035	0.7845	0.0026
28	0.9708	0.0059	0.7869	0.0050

**Table 13:** The mean and normalized area under the learning curves represents the contributions and the combinations of each component in the survey–identify–reinforce–optimize framework.

Improvement	Winning Rate		Win Efficiency	
	Mean	Standard Deviation	Mean	Standard Deviation
Survey only	0.5904	0.0212	0.3255	0.0286
Identify only	0.9652	0.0069	0.8039	0.0018
Reinforce only	0.6658	0.0453	0.4378	0.0525
Optimize only	0.6379	0.0190	0.3794	0.0310
Survey and identify	0.9551	0.0120	0.8136	0.0072
Survey, identify, and reinforce	0.9616	0.0082	0.8329	0.0094
Survey, identify, reinforce, and optimize	0.9655	0.0039	0.8226	0.0057

**Table 14:** The mean and normalized area under the learning curves represents the contributions on the scales used for the optimized rewards and penalties.

Optimized Reinforcement	Winning Rate		Win Efficiency	
	Mean	Standard Deviation	Mean	Standard Deviation
block_scale only	0.9595	0.0056	0.8237	0.0029
progress_scale only	0.9466	0.0020	0.8206	0.0010
presence_scale only	0.9577	0.0033	0.8246	0.0070
action_efficiency only	0.9540	0.0083	0.8226	0.0070
Enabled all reinforcements	0.9655	0.0039	0.8226	0.0057

## Data Availability

The original data presented in this study are openly available in GitHub at: https://github.com/yamaguchishingo/siro_mdpi2026 (accessed on 8 April 2026).

## Abbreviations

    The following abbreviations are used in this manuscript:

IoT	Internet of Things
WSN	Wireless sensor network
DDoS	Distributed denial of service
BDS	Botnet Defense System
ML	Machine learning
SL	Supervised learning
RL	Reinforcement learning
SIRO	Survey–identify–reinforce–optimize
CNN	Convolutional neural network
Conv2D	2D convolutional layer
AULC	Area under the learning curve
GNN	Graph neural network
BFS	Breadth-first search

## Appendix A

To simulate the propagation of botnet and white-hat worm infection, Algorithm A1 utilizes the manhattan_coordinate() function to determine the vertically and horizontally neighboring device coordinates to be scanned to see if the devices are infectable or not. The manhattan_coordinate() implementation is shown in Algorithm A2.

**Algorithm A1** Simulate the propagation of botnet and white-hat worm infection.
Input: Device resiliency resilience_map, infection state infection_map, *m*, *n* Output: Updated infection state $infection\_map^{\prime \prime }$ 1: simulate_infection(resilience_map,infection_map,m,n) 2: Copy the infection state: $infection\_map^{\prime }\leftarrow infection\_map$ 3: // Simulate the botnet infection first 4: **for** i=0 to m−1 **do** 5: **for** j=0 to n−1 **do** 6: **if** infection_map[i,j]=1 **do** 7: Get neighbors: coordinates←manhattan_coordinate (i,j,m,n) 8: **for** dx,dy **in** coordinates **do** 9: **if** resilience_map[dx,dy]<0.5 **and** infection_map[dx,dy]=0 **do** 10: Insert botnet: $infection\_map^{\prime }\left[dx,dy\right]\leftarrow 1$ 11: **end for** 12: **end for** 13: **end for** 14: Copy the infection state: $infection\_map^{\prime \prime }\leftarrow infection\_map^{\prime }$ 15: // Followed by the white-hat worm infection 16: **for** i=0 to m−1 **do** 17: **for** j=0 to n−1 **do** 18: **if** $infection\_map^{\prime }\left[i,j\right]=2$ **do** 19: Get neighbors: coordinates←manhattan_coordinate (i,j,m,n) 20: **for** dx,dy **in** coordinates **do** 21: **if** resilience_map[dx,dy]<0.5 **do** 22: **if** $infection\_map^{\prime }\left[dx,dy\right]=0$ **or** $infection\_map^{\prime }\left[dx,dy\right]=1$ **do** 23: Insert white-hat worm: $infection\_map^{\prime \prime }\left[dx,dy\right]\leftarrow 2$ 24: **end for** 25: **end for** 26: **end for** 27: **return** $infection\_map^{\prime \prime }$ 28: **End Function**

**Algorithm A2** Find the neighboring coordinates with Manhattan distance of 1.
Input: *x*, *y*, *m*, *n* {0≤x<m, 0≤y<n} Output: Neighboring coordinates coordinates 1: manhattan_coordinate (x,y,m,n) 2: Initialize list: coordinates←[] 3: **if** x+1<m **then** 4: // Scan neighboring coordinates 5: coordinates.append((x+1,y)) // Right 6: **if** x−1≥0 **then** 7: coordinates.append((x−1,y)) // Left 8: **if** y+1<n **then** 9: coordinates.append((x,y+1)) // Up 10: **if** y−1≥0 **then** 11: coordinates.append((x,y−1)) // Down 12: **return** coordinates 13: **End Function**

To create a mask that reduces discrete action space complexity and enables the agent to explore various winning strategies, Algorithm A3 uses the chebyshev_coordinate() function to identify the device coordinates that are vertically, horizontally, and diagonally neighboring to the infected devices.

The chebyshev_coordinate() function in Algorithm A4 is an extended version of Algorithm A2. The only difference is the additional conditionals added in lines 12 to 20, which ensure that the diagonally neighboring devices are both empty and indefectible. By unmasking the diagonally neighboring devices, the RL could efficiently block two infection routes at the same time.

**Algorithm A3** Use Chebyshev distance of 1 from the infected devices to create the mask.
Input: Current infection state $infection\_map_{t}$, resilience_map, *m*, *n* Output: Masked action space masked_map 1: chebyshev_mask ($resilience\_map,infection\_map_{t},m,n$) 2: Initialize preapplied masked action space: masked_map[m][n]←False 3: // Scan for the infected devices 4: **for** i=0 to m−1 **do** 5: **for** j=0 to n−1 **do** 6: **if** $infection\_map_{t}\left[i,j\right]=1$ **do** 7: Get neighbors: coordinates←chebyshev_coordinate (i,j,m,n) 8: **for** dx,dy **in** coordinates **do** 9: **if** resilience_map[dx,dy]<0.5 **and** $infection\_map_{t}\left[dx,dy\right]=0$ **do** 10: Remove mask: masked_map[dx,dy]←True 11: **end for** 12: **end for** 13: **end for** 14: **return** masked_map 15: **End Function**

**Algorithm A4** Find the neighboring coordinates with Chebyshev distance of 1.
Input: *x*, *y*, *m*, *n* Output: Neighboring coordinates list coordinates 1: chebyshev_coordinate (x,y,m,n) 2: Initialize list to store the coordinates: coordinates←[] 3: // Generate vertically and horizontally neighboring coordinates 4: **if** 0≤x+1<m **do** 5: Right: coordinates.append((x+1,y)) 6: **if** 0≤x−1<m **do** 7: Left: coordinates.append((x−1,y)) 8: **if** 0≤y+1<n **do** 9: Up: coordinates.append((x,y+1)) 10: **if** 0≤y−1<n **do** 11: Down: coordinates.append((x,y−1)) 12: // Generate diagonally neighboring coordinates 13: **if** 0≤x+1<m **and** 0≤y+1<n **do** 14: Right-up: coordinates.append((x+1,y+1)) 15: **if** 0≤x+1<m **and** 0≤y−1<n **do** 16: Right-down: coordinates.append((x+1,y−1)) 17: **if** 0≤x−1<m **and** 0≤y+1<n **do** 18: Left-up: coordinates.append((x−1,y+1)) 19: **if** 0≤x−1<m **and** 0≤y−1<n **do** 20: Left-down: coordinates.append((x−1,y−1)) 21: **return** coordinates 22: **End Function**

## Appendix B

The rewards and penalties of the previous RL-based BDS launcher proposed by Yoshioka et al. [16] are designed to optimize the insertion of white-hat worms while using masked illegal moves and single tensor input. Appendix B discussed the implementations used in Section 4.2.

Compared to the episodic reward proposed by the SIRO framework in Equation (7), the episodic reward used by the previous RL-based BDS launcher utilized static values for both winning and losing the simulation, as shown in Equation (A1). While it is simple, the agents must rely on step-based rewards to know how well they perform during the simulations.

(A1) $$terminal_{old}=\left\{\begin{array}{c} \begin{array}{cc} \mathrm{win} & =1\\ \mathrm{lose} & =-1 \end{array} \end{array}\right.$$

For the step-based rewards, the previous RL-based BDS launcher utilized three key principles to optimize the white-hat insertion under:

1. Placement distance reward;
2. Edge closeness penalty;
3. Path-blocking reward.

The placement distance reward aims to reinforce the agents to insert the white-hat worms as close as possible to the botnet. It works by calculating the shortest distance $d_{shortest}$ from the newly placed white-hat worm and the set of botnets coordinates before normalizing and using it as the reward, as shown in Equation (A2).

(A2) $$distance=0.3\left(1-\frac{d_{shortest}}{m\cdot n}\right)\;\mathrm{where}\;d_{shortest}=\min_{bot\in botnets}\left(\left|x-x_{b}\right|+\left|y-y_{b}\right|\right)$$

Meanwhile, the edge closeness penalty aims to discourage the agents from letting the botnets move closer to the network boundaries. For each botnet coordinate $\left(i,j\right)$, the distance to the closest boundary is calculated in Equation (A3) to obtain $R_{edge}$. Then, the average is calculated and multiplied by the penalty scale in Equation (A4).

(A3) $$\begin{array}{c} R_{edge}=\frac{1}{d_{edge}-1}\;\mathrm{where}\;d_{\mathrm{edge}}=\min\left\{\begin{array}{cc} m-1-j, & (\mathrm{right}\;\mathrm{boundary}),\\ j, & (\mathrm{left}\;\mathrm{boundary}),\\ i, & (\mathrm{top}\;\mathrm{boundary}),\\ n-1-i, & (\mathrm{bottom}\;\mathrm{boundary}). \end{array}\right. \end{array}$$

(A4) $$closeness=-0.1\cdot \frac{1}{botnet\_count_{t}}\sum_{count}R_{edge}^{count}$$

Finally, the path-blocking reward aims to reinforce the agents to block the shortest traversable path to the boundary. With $L_{\mathrm{prior}}$ and $L_{\mathrm{after}}$ representing the path length prior to and after the white-hat worm insertion, the path-blocking reward is calculated as shown in Equation (A5).

(A5) $$traverse=0.5\left(L_{\mathrm{after}}-L_{\mathrm{prior}}\right)$$

The total reward given at the end of each step is shown in Equation (A6), where the episodic reward is only given when the simulation reaches its terminal state.

(A6) $$total\_reward_{old}=distance+closeness+traverse+\left\{\begin{array}{cc} terminal_{old}, & \mathrm{if}\;\mathrm{terminal}\\ 0, & \mathrm{otherwise} \end{array}\right.$$

## Appendix C

Figure A1 shows that the SIRO framework reduced the step-to-win to within three steps, with the DQN-based agent having the lowest step-to-win shown in Table A1.

**Table A1:** The highest winning rates achievable by each reinforcement learning and their step-to-win metrics.

Algorithm	Implementation	Highest Winning Rate [%]	Step-to-Win
Vanilla policy gradient	Previous	79	3.9114
	Improved	99	2.3030
Deep Q-network	Previous	79	3.4177
	Improved	100	2.3500
Proximal policy optimization	Previous	77	5.4026
	Improved	100	2.3600
MuZero	Previous	71	3.9718
	Improved	97	2.6289
